# The Black American experience: Answering the global challenge of broadening participation in STEM/agriculture

**DOI:** 10.1093/plcell/koae002

**Published:** 2024-01-29

**Authors:** Eduardo Haverroth, Mariah Gobble, LaTosha Bradley, Kailyn Harris-Gilliam, Alicia Fischer, Cranos Williams, Terri Long, Rosangela Sozzani

**Affiliations:** Department of Plant and Microbial Biology, North Carolina State University, Raleigh, NC 27607, USA; Department of Plant and Microbial Biology, North Carolina State University, Raleigh, NC 27607, USA; CALS Office of Diversity and Inclusion, North Carolina State University, Raleigh, NC 27695, USA; Science and Technologies for Phosphorus Sustainability Center (STEPS), North Carolina State University, Raleigh, NC 27606, USA; Friday Institute for Educational Innovation, College of Education, North Carolina State University, Raleigh, NC 27606, USA; Department of Electrical and Computer Engineering, North Carolina State University, Raleigh, NC 27606, USA; Department of Plant and Microbial Biology, North Carolina State University, Raleigh, NC 27607, USA; Department of Plant and Microbial Biology, North Carolina State University, Raleigh, NC 27607, USA

Dear Editor,

Since the abolition of slavery in the United States, marked by the passing of the 13th Amendment in 1865, our nation has made significant strides toward constructing an inclusive and equitable society. The brutality of the American chattel slavery stands as a stark reminder of the progress achieved and the profound lessons to be gleaned from history. Despite notable advancements, the enduring impacts of slavery and its aftermath necessitate a continued commitment to fostering equality. This journey toward justice remains incomplete, with systemic issues such as Jim Crow laws, education segregation, redlining, and employment discrimination persisting.

To confront these historical injustices, it is imperative to implement inclusive policies and programs. By acknowledging and addressing the repercussions of slavery, society can dismantle barriers that hinder equal opportunities. This letter specifically addresses the underrepresentation of Black Americans and people of color in Science, Technology, Engineering and Mathematics (STEM) fields, particularly plant and agricultural science. It introduces an innovative approach, the Accelerate Integration of Engineering, Plant Science, and Agricultural Research using Artificial Intelligence (AI2EAR) network, a crucial component of the National Science Foundation’s (NSF) Accelerating Research through International Network-to-Network Collaborations program (AccelNet).

Achieving a truly inclusive society requires not only attracting Black community members to STEM fields but also preparing them for leadership roles and fostering a genuine sense of belonging. This letter aims to amplify the impact of AI2EAR and inspire the broader STEM community to adopt new and impactful diversity, equity, and inclusion (DEI) initiatives. By promoting meaningful reflection, understanding, and action, we aspire to contribute to the diversification of STEM fields. Advocacy for programs like AI2EAR, which includes programming designed to support underrepresented minorities, can catalyze the creation of a more diverse and inclusive future in STEM and agriculture.

## Black scientists in agriculture and plant science: A historical perspective

The consequences of policies and practices from the slavery era affect economic opportunities even 160 yr later. For example, the Homestead Act and the Morrill Land Grant Act, both enacted in 1862 before the 14th Amendment formerly recognized enslaved people as citizens, continue to cast long shadows, impeding Black Americans’ full participation in the land market and hindering the creation of generational wealth (Smith 2004; Aminetzah et al. 2021). The historic lack of education about legal protections such as wills that facilitate property transfer to the next generation, and discriminatory lending practices, compound these challenges (Carter and Alexander 2020). This exclusion from property ownership and the resultant absence of generational wealth parallel the ongoing lack of representation in STEM fields that persist today. In addition, for many Black Americans, the significant contributions of their ancestors often go unrecognized, leading to feelings of disconnection from the land they once cultivated and nurtured. Today, underrepresented minorities represent 42% of the US population. However, they make up only 30% of full-time STEM/Agriculture employees, 7% of STEM workers overall, and 5% of farmers (NSF 2019). These statistics underscore a significant disparity, which will only intensify in coming years as this population increases to a predicted 57% by 2060 (U.S. Census Bureau 2017).

**Figure 1.**
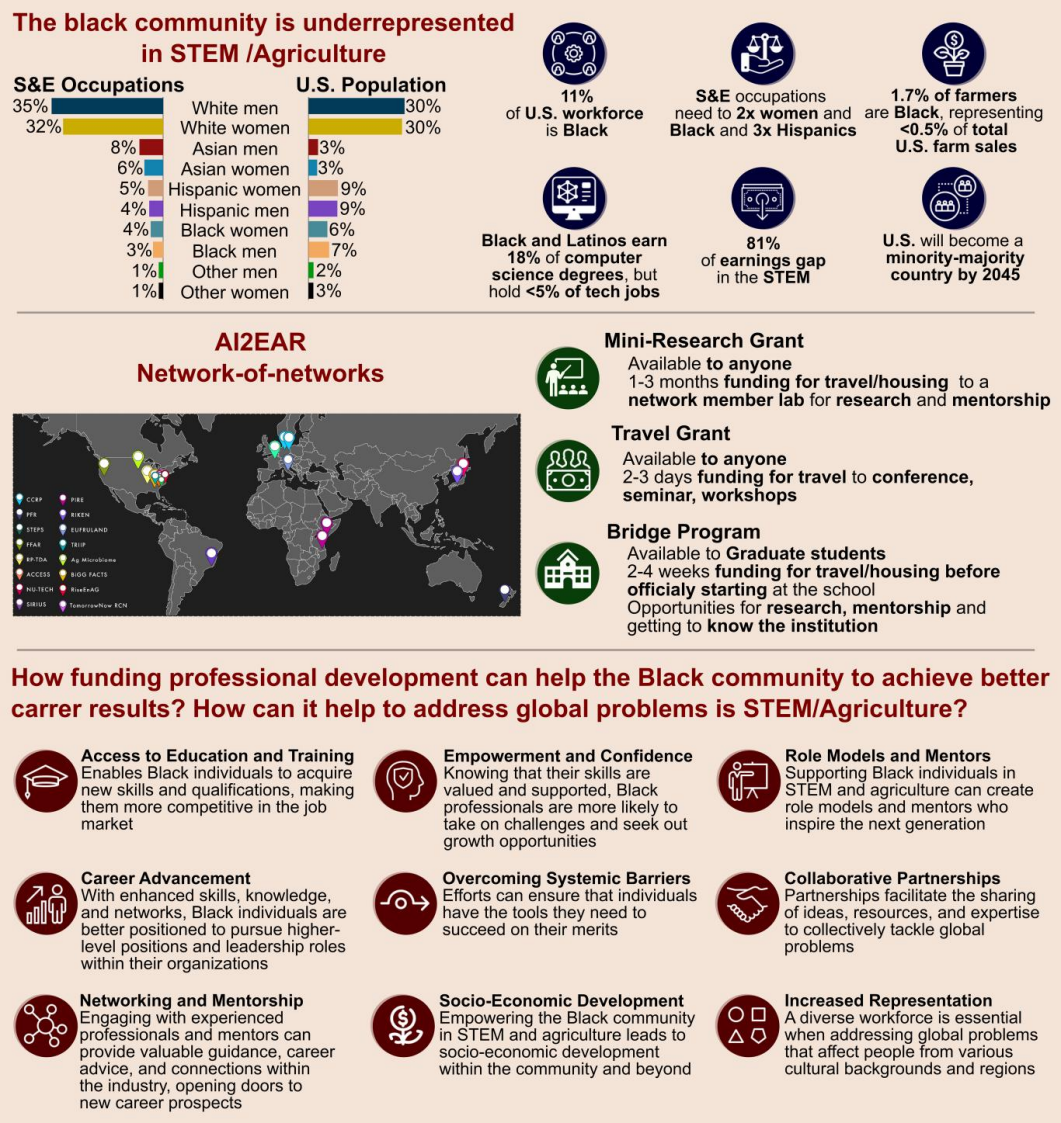
The Black community faces underrepresentation in STEM/Agriculture, holding fewer positions than its proportional population, and experiencing a significant earnings gap. AI2EAR, a network comprising 17 international networks, shares the common goal of advancing the STEM/Agriculture fields. AI2EAR focuses on the professional development of early career researchers, students, and other professionals in these fields, advocating for increased diversification to authentically meet the demands of food supply and drive societal change. Providing funding for professional development initiatives within the Black community empowers individuals, allowing them to surmount barriers and attain improved career outcomes. This not only benefits the individuals directly involved but also cultivates a more diverse, inclusive, and thriving workforce, broadening perspectives to effectively tackle global challenges. The percentages in the upper panel are from the NSF 2019, NSF 2017, U.S. Census Bureau 2017, USDA 2017 and Fry, Kennedy and Funk 2021.

The underrepresentation of minorities and women, particularly in computing, contributes to a data science workforce shortage (Cahill et al. 2022). Black Americans and Latinos earn 18% of computer science degrees but hold just 5% of tech jobs (NSF 2019), while Black engineers constitute only 3.3% of the engineering community (NSF 2017). In agriculture, Black farmers represent a mere 1.7%, reflecting <0.5% of total US farm sales (USDA 2017). This stark underrepresentation highlights historical issues within the American workforce, shaping the nation’s agricultural infrastructure, food systems, and technological advancements predominantly through the lens of the White-dominated STEM/Agriculture fields (Fig. 1; NSB 2020; Madzima and MacIntosh 2021; Montgomery and Whittaker 2022).

The experience of Black individuals in Engineering, Agricultural Research, and Artificial Intelligence is marked by profound underrepresentation, leading to systemic exclusion and inequity (Kulis et al. 2000; Strayhorn et al. 2013; Morris and Washington 2017; McGee 2020). Addressing these challenges must extend beyond individual experiences to tackle broader community-level inequities. Failure to address these issues directly through appropriate policies and programs risks depriving the United States of the manifold benefits that could arise from a greater inclusion of the Black community in these disciplines. Raising awareness of successful career outcomes becomes crucial, countering negative perceptions of STEM/Agricultural fields among Black Americans (Jones 2007). Without this awareness, statistical trends indicating underrepresentation persist, hindering the realization of financial stability, health, and freedom through careers in plant science, agriculture, and engineering (Fig. 1; Morris and Washington 2017; Madzima and MacIntosh 2021). Recruitment initiatives within higher education and research institutions emerge as a promising avenue for raising awareness. Programs with a proven track record, such as the Meyerhoff Scholarship Program at the University of Maryland, have effectively nurtured Black students into scientists, providing crucial financial assistance and fostering a sense of belonging (Stolle-McAllister et al. 2011). A pressing requirement exists for similar initiatives across other land grant institutions, particularly in less-explored STEM and Agriculture domains such as mathematical modeling and Artificial Intelligence. Notably, Friesner et al. (2021) have developed a comprehensive resource aimed at engaging and attracting a more diverse student body in plant sciences.

Recognizing the imperative for sustained mentorship and support, especially for underrepresented populations, there is a continued need to champion pipeline programs and initiatives. These endeavors are essential for enhancing professional qualifications within the Black community, ultimately paving the way for successful career outcomes. Advocacy for such programs aligns with the broader goal of fostering diversity and inclusivity in the STEM and Agriculture fields, acknowledging that a proactive and sustained approach is vital for addressing historical disparities and promoting lasting change.

## Diversity in agriculture and plant science: An international perspective

Clearly, the United States continues to wrestle with the impact of slavery. Outside the United States, however, many perceive that the need for DEI initiatives is merely an “American issue” (Luthra 2022). To gain insight into this question, we recently gathered international perspectives on DEI initiatives during the 2023 International Conference on Arabidopsis Research in Chiba, Japan, and through interviews with the members of the NSF AccelNet, AI2EAR network. These interviews suggest that the global vision for DEI is characterized by diverse viewpoints, often influenced by variations in the advancement of DEI policies across countries and the specific target groups of these policies (Delaine et al. 2016; Barroso et al. 2022). A prevailing sentiment from those interviewed is that historically, DEI efforts have been politically motivated and frequently exclude members of underrepresented groups from meaningful dialogue (Long and Mejia 2016; Morris and Washington 2017; Madzima and MacIntosh 2021). Consequently, a notable challenge lies in engaging those in positions of influence, typically belonging to the majority, and helping them recognize the innovation and societal advancement benefits associated with robust DEI efforts.

Similar to other international groups (Claeys-Kulik et al. 2019; Luthra 2022; Vanthof et al. 2023), our interviewees commonly expressed the view that investing in minority communities and promoting awareness within society are integral to the successful integration of these communities into all facets of society. However, due to historic barriers that have restricted access to certain societal and educational spaces, people from underrepresented groups are oftentimes unaware of available educational and professional opportunities. Therefore, for a society to genuinely embody inclusivity, intentional efforts must cultivate a sense of belonging, and information about opportunities must be effectively communicated.

In this context, pipeline programs offering mentorship to Black students and other minorities in select US universities have demonstrated promising results (Brunsma et al. 2016; Atkins et al. 2020; Samari et al. 2022; Grilo et al. 2023). Such initiatives, which focus on introducing undergraduate students to higher education and creating a supportive environment that fosters a sense of belonging, constitute an exceptional strategy for cultivating highly qualified individuals within the Black community. Furthermore, initiatives concentrating on enhancing professional qualifications and fostering interdisciplinary networks within the Black community can significantly propel the leadership of Black students, positioning them for decision-making roles in STEM and agriculture fields.

## AI2EAR: A panoramic approach to engaging underrepresented minorities in agriculture and STEM

The recently established NSF AccelNet, AI2EAR, constitutes a network of 17 international networks united in the shared objective of advancing STEM/Agriculture fields through collaborative data and knowledge sharing to ensure sustainable food production and global food security. Research labs within the program are conceived as open spaces with multiple principal investigators, allowing students to seek mentorship from a range of researchers, in addition to their primary strategically paired mentors based on research interests. Multiple programs within AI2EAR are designed to fund and support the career growth of underrepresented minority students and researchers (https://www.ai2ear.org/networks). In support of historically marginalized community members, AI2EAR is implementing programming components offering personalized advising and connecting students with multiple mentors, including diverse peers, professionals, and various professional development programs.

AI2EAR recently piloted a graduate bridge program at NC State, which enabled 3 incoming graduate students from underrepresented groups to be on campus 2–4 wk before regular classes commenced, facilitating personalized meetings with advisors, orientation to campus resources, and early engagement in research alongside mentors. This program enabled each student more readily transition from undergraduate to graduate school, where they all continue to thrive in their respective programs. Additionally, undergraduate, graduate, and postdoctoral students and assistant professors who are seeking to engage with the international AI2EAR collaborative community have the opportunity to apply for an AI2EAR mini-grant to support short-term travel to conferences, workshops, and seminars, as well as long-term travel to visit laboratories within the 17 networks. This program enables exposure to new techniques and fosters global collaborations. Consequently, early career researchers not only gain opportunities for research, networking, and mentor support but also develop essential “soft skills” such as cross-cultural communication, time management, problem-solving, and leadership, equipping them for continued success beyond the program’s conclusion. To date, 7 graduate students, postdocs, and assistant professors have participated in this pilot program, attending and presenting their work in international conferences and workshops in Japan, Brazil, and the United States. Over half of these participants are from underrepresented groups, and for several, this program offered their first opportunity for international travel or oral presentations of their work. Additionally, new multi-institution collaborative projects arose from these travels, which enabled scholars to keep receiving mentorship from a diverse range of experts from different regions of the globe. Further program activities involve the organization of workshops during conferences. The workshop themes cover a range of subjects, including Engineering, Plant Sciences, Agriculture, Artificial Intelligence, and Diversity and Inclusion in Science.

The pervasive lack of diversity in Agriculture and Engineering presents formidable challenges in addressing global food security. It is imperative to dismantle barriers to inclusivity, particularly those rooted in historical inequities and perpetuated by existing programs and policies. Recognizing the integral role of historically disadvantaged communities, such as the Black American community, in global prosperity, AI2EAR asserts that efforts to involve them in decision-making regarding global challenges should be a paramount priority. In addition to its existing travel funding programs, AI2EAR plans to continue to work to tackle accessibility barriers through recorded and hybrid-virtual STEM and agriculture meetings, as well as online access to professional development training, mitigating travel limitations.

In essence, initiatives like AI2EAR are poised to introduce the much-needed diversity among seasoned professionals advocating for policy change, future professors, and other leaders actively researching to advance these fields through national and international collaborative knowledge sharing. Engaging underrepresented minorities in these efforts will drive innovation in developing a sustainable food supply, address the challenges of climate change, and provide role models for the next generation of students aspiring to leadership roles in these fields, contributing to sustained diversification for generations to come.

## Data Availability

The data underlying this article will be shared on reasonable request to the corresponding author.

## Dive Curated Terms

The following phenotypic, genotypic, and functional terms are of significance to the work described in this paper:

NSF Gramene: AT4G04910
NSF Araport: AT4G04910
